# Challenges and Enablers for Smartphone Use by Persons With Vision Loss During the COVID-19 Pandemic: A Report of Two Case Studies

**DOI:** 10.3389/fpubh.2022.912460

**Published:** 2022-07-06

**Authors:** Suraj Singh Senjam, Susan A. Primo

**Affiliations:** ^1^Department of Community Ophthalmology, Dr. R. P. Centre for Ophthalmic Sciences, All India Institute of Medical Sciences, New Delhi, India; ^2^Department of Ophthalmology, Emory University School of Medicine, Atlanta, GA, United States

**Keywords:** visual impairment, assistive technology, smartphone use, accessible apps, challenges, enablers

## Abstract

**Purpose:**

Studies have reported that knowledge and skills to operate smartphones among people with profound visual loss are limited especially in low- to middle-income countries as many important functions of smartphones are unknown to them. This report presents smartphone use, its challenges, and enablers in two persons with profound visual impairment while executing their daily routine and instrumental living activities amidst the COVID-19 pandemic.

**Case selection and interview:**

During the lockdown period, we provided tele (vision) rehabilitation service. From the list of the callers, we purposely selected two callers with significant visual impairment, one woman and one man, to allow us to gather rich information related to smartphone use, enablers, and challenges faced during the usage. A semistructured interview was done to obtain insights into the information. The selection criteria were (1) continuous smartphone use independently for more than 5 years; (2) graduation-level education or higher; and (3) no additional disabilities.

**Discussion:**

We found substantial use of smartphones in executing their daily and instrumental daily living activities by these two participants. The extent of the use of mainstream apps for various tasks was almost equivalent to what we observed among sighted persons. The most important enabling factors were the presence of a screen reader “TalkBack” on Android phones and data connection of the mobile, followed by the ability to assess multiple languages using the text-to-speech feature. A supportive environment from peers or family members is important for the beginner. Poor battery backup, frequent unwanted ads or pop-ups while using the phone, not readable contents with a screen reader, e.g., CAPTCHA, and slow or unresponsiveness of the screen reader were frequent challenges faced by them. Both cases reported that around 80% of daily solutions were helped by using a smartphone.

**Conclusions:**

The current advances in accessible technology of smartphones enable an individual with profound visual loss to use them almost equivalently as a sighted person. To reduce the gap in digital inclusion, people with visual impairment should be encouraged to use the smartphone for their daily solutions with attention to proper training.

## Introduction

Smartphones are not only increasingly ubiquitous in low- to middle-income countries (LMICs) but also have become one of the essential commodities in life for everyone. Earlier, users with visual impairment face substantial challenges in terms of accessing and inclusion in the smartphone environment despite a widespread prevalence of touch screen technology. With the progress in screen readers software technology with audio or tactile feedback, users with visual impairment do not need sighted assistance to be used independently. Today, mobile technologies have grown exponentially over the past few years, including built-in accessible features and third-party accessible applications for people who are visually impaired (1). Such a recent advance in smartphone technology provides a new opportunity to bring various solutions in performing everyday activities for persons with visual disabilities. Evidence shows that the use of smartphones enhances the quality of life of people with visual impairment by improving their autonomy and safety, and encouraging them to interact with the community and society (2, 3). Various studies have reported the use of smartphones among blind users for social media engagement, performing instrumental daily living activities such as shopping, finance management, navigation, educational purposes, and employment (4–6).

As of today, the majority of the evidence on smartphone use among people with visual impairment IS documented primarily from high-income nations as compared to LMICs though few studies reported smartphones use particularly in students with visual impairment (4, 7, 8). Nearly a decade ago, smartphones were associated with a high cost and were considered to be an electronic product for people living in high-income countries. However, the cost of smartphones has decreased over the past few years, the availability of smartphones across LMICs is also growing (9). Access to smartphones, therefore, is not a major issue in these countries. Although smartphone use is documented among people with visual impairment for their daily solutions in LMICs, there is a lack of evidence about how much a smartphone is helpful in terms of executing tasks for independent daily and instrumental daily living activities of a person with profound visual impairment or total blindness. A study in Nepal reported a lack of knowledge and skills to operate the accessible features and apps of smartphones among blind students despite the participants being aware of smartphones and their accessibility features (10). Several studies also reported the inability to fully use the accessible features, and many vital functions of smartphones were undiscovered among blind users (10, 11). Therefore, there is a need for documentation for rich information on comprehensive usage of the smartphone along with various challenges, particularly by people with profound vision impairment or total blindness who have an adequate user experience.

Given the current advance in digital technology, there may be a potential risk that persons with visual disabilities might be left behind without availing of the full use of such accessible technology, especially in LMICs. The inclusion of people with visual disabilities in the digital world is essential for their overall development and quality of life. A smartphone is one of the digital technologies that can support social inclusion, connectedness, and participation in civic life (12, 13). In addition, the smartphone is one of the known assistive technologies (ATs) under the World Health Organization (WHO) Global Cooperation on Assistive Technology that helps to reduce functional difficulties (14). The GATE aims to improve access to AT and its related services as a part of the Universal Health Coverage. Besides, the Sustainable Development Goals focus on the need for social inclusion and participation and pledged that no one should be left behind in all dimensions of life based on disabilities or any other personnel disadvantages or characteristics (15). Therefore, generating evidence on the smartphone's usability among blind users is critically important which will further help not only digital inclusion but also social inclusion and participation in the current digital world. Indeed, studies reported smartphone use for social media engagement actively among blind users which is directly linked to an increase in social inclusion and participation (11, 12, 16).

In addition, to our knowledge, the majority of individuals with visual disabilities in LMICs still depend on a simple basic or feature phone which has limited functionality compared to the current smartphones. Moreover, mobile technology is pragmatic, cost-effective, and universally designed assistive devices, so it is less likely to be a social stigma and make public attention among blind users. Therefore, there is a need to promote the use of smartphones across the population with visual disabilities, especially in LMICs.

The purpose of this case report was to understand the extent of smartphone usage by persons with profound visual impairment to the maximum. It also presents the challenges and enablers of smartphone use by them during the COVID-19 pandemic in Delhi, India. Such a case study will provide a positive impression that will motivate smartphone use among the visually impaired, thereby helping to address the digital inclusion gap of persons with visual disabilities. At the same time, it will also help to assess whether the visually disabled are one of the potential clients of the current smartphone world.

## Case Selection

We run a vision rehabilitation (VR) clinic where patients with low vision and blindness are referred from various ocular subspecialties of the out-patient department of our eye center and from other facilities or organizations. To maintain the continuum of services, we also liaised with many civil or community-based organizations, including schools for the blind in Delhi and National Capital Region. During the COVID-19 emergency period in 2021, we circulated a few telephone hotline numbers to the representatives of these organizations for support of healthcare and rehabilitation services.

The cases were selected purposely from the list of callers who have availed of VR services to date. Graduate-level and above in education, profound visual loss, active users of smartphones for more than 5 years independently, and no other co-disabilities were selection criteria for the cases. The VR staff contacted the participants by telephone and scheduled them for an in-person meeting at the clinic. We aimed to collect rich information on the use of smartphones by persons with total or profound visual loss or not relying on vision functions for smartphone use.

## Case Description

### Case 1

The patient, named UK, was a man aged 29 years with total blindness (no light perception in both eyes), diagnosed with phthisis bulbi with glaucoma and has been using a smartphone independently for the last 10 years. He has no other co-disabilities. He completed his post-graduation 2 years ago and currently looking for a suitable job. He understands the English language but cannot speak it fluently. His degree of disability as shown on his certificate was 100%.

### Case 2

The patient, named JS, was a woman aged 27 years with profound vision loss with her left eye vision no light perception and finger counting close to the face in the right eye. She was an employee in a private bank. She has been using smartphones independently for more than seven years. She was a case of Stevens–Johnson Syndrome with corneal opacity. She has no other disabilities. She understands the English language but cannot speak fluently. Her degree of disability as shown on her certificate was 90%.

## Questionnaire and Interview

A face-to-face, in-depth interview with the cases was conducted using a semistructured questionnaire to obtain insight into the spectrum of the use of smartphones, enabling factors, and various challenges encountered by the users. The purpose of using the personal interview was to gain significant information of insights and to improve data accuracy.

Section A of the questionnaire consisted of two parts, namely, the domains of smartphone use and the enabling factors for each domain of the usage. Section B consists of additional factors that help to use smartphones consistently. The last Section C part includes challenges faced by cases while using the smartphone. The responses to the last two sections of the questionnaire were ordinal. The questionnaire was developed in consultation with the trainers who provide training, primarily on the use of digital assistive devices for visual impairment. Furthermore, pretesting was done among non-study individuals with similar characteristics. The reason for conducting several pretests was we expected that respondents will have different levels of information and experiences in using a smartphone, whereby, the question developed could capture adequate information on the use of smartphones at the maximum level. One open-ended question was included “How much does your smartphone help in doing daily living activities without support from sighted persons? Rate it in percentages from 0 to 100.”

We used the English language for the tool since the interviewer was well-versed in English. The day of the interview was fixed according to the convenience of the cases. The cases were explained about the purpose and the content of the questionnaires before we obtained informed written consent. The response to the entire questionnaire took ~60 min of participants' time.

The rehabilitation team helped both cases in terms of the issuance of a visual disability certificate from the institute. Case number one was informed about various relevant schemes for his benefits, including job reservation under the Government of India. He was educated about available vocational training facilities in Delhi and National Capital Region that we liaised with our VR clinic and was advised to contact them in the future if he is willing to undergo any vocational training in our networking centers. Besides these, they were provided the orientation and mobility training as per standard guidelines and standard dining techniques and explained the need for the creation of a safe environment, particularly at home and the workplace. They were educated about COVID-19 preventive steps and informed to contact for any medical conditions, including COVID-19-related issues.

## Discussion

The present case study aimed to explore comprehensively the use of the smartphone, including challenges, while executing daily and instrumental daily living activities by visually disabled persons who do not rely on vision for use. We believe that the diagnosis of blinding eyes will not impact the study findings. However, we recorded the diagnosis and percentages of visual disability written on the certificate which is issued based on the guidelines given by the Ministry of Social Justice Empowerment, Government of India.

This report shows that not only does the current smartphone technology provide an invaluable asset to everyone but the recent advancement in touchscreen and accessible features and apps means the content has become more accessible to people with visual disabilities. In this case report, we presented two cases with profound visual impairment who have been using smartphones for more than 5 years in executing and bringing solutions for their daily activities. This report will provide rich information regarding the extent of smartphone use by persons with severe or total blindness. Besides this, the report may motivate other people with similar visual problems, particularly from LMICs, to adopt smartphones in their daily living lives.

## Usage of Smartphones

The domain of smartphone usage by two of them is as comprehensive as smartphone use by a sighted person. The extent of smartphone use is quite substantial in bringing solutions and improving other daily living activities. Table 1 shows the spectrum of smartphone use and means for each usage by the cases. The majority of routine tasks were executed with the help of available mainstream support systems or means, for example, they used SMS (short message services), voice calls, audio-visual calls, and emails for communication with friends and family members. Likewise, YouTube, Hot star, Google Pay, Paytm, Amazon prime, Flip Kart, and Zomato apps were being used for various daily solutions. Furthermore, Facebook, WhatsApp, Instagram, and even Twitter were also used for social media engagement. In addition, participants also used special accessible apps for their daily tasks: Insta Reader, Envision AI accessible app were used for reading print materials, Mani App for currency identification, and Eye D pro for color identification. Furthermore, OLA (Operational Level Agreement) and UBER (Uncorrected Bit Error Rate) apps were mainstream apps that were used for online booking of local travel.

**Table 1:** Domains of smartphone use by person with profound visual impairment.

**Domains of smartphone use**	**Available means to use the domain**	**Case 1**	**Case 2**
Communication	SMS, Voice call, Audio-visual call, email, any other	SMS, Voice calls, Audio-visual, email	SMS, Voice calls, Audio-visual
Health consultation	SMS, Voice call, Audio-visual call, any other	Voice calls	Voice calls
Information on COVID-19	Newspaper, YouTube, News channel, any other	YouTube, News Channel	YouTube, Twitter
Online banking	Mainstream system, special apps	Not used	Not used
Online payment	Mainstream apps, special apps	Google Pay, Phone Pay, Paytm,	Google Pay, Paytm,
Online shopping/Tele shopping	Mainstream apps, special apps	Flip Kart, Amazon prime, Zomato	Flip Kart, Amazon prime, Swiggy, Meesho
Reading print materials	Special apps	Insta reader	Envision AI app
Entertainment	DISH satellite TV, YouTube, Netflix, Amazon prime,	YouTube, Tiktok, Wynk, Hotstar	YouTube, Jio TV
Color identification	Special accessible apps	Eye D pro	Not used
Money identification	Special accessible apps	Mani App, Mani reader	Mani App, KITNA App
Object identification	Special accessible apps	Not used	Not used
Online booking for local travel	Mainstream apps, special apps	OLA, UBER app	OLA, UBER app
Indoor navigation	Special apps	Not used	Not used
Identifying medicine	Special app	Not used	Envision AI
Alarm/reminder	Mainstream system, special apps	Mobile alarm	Mobile alarm
Audio-video conferencing platform	Mainstream system, special apps	Google meet, Zoom	Google meet, Zoom, CISCO
Calculator	Mobile calculator, special apps	Mobile calculator	Mobile calculator
Face recognition	Special apps	Not used	Not used
Social media	Mainstream system, special apps	Facebook, WhatsApp, Instagram, telegram	Facebook, WhatsApp
Photography	Mainstream camera, Special apps	Documents only	Mobile camera,
Data storage and recording	Mainstream system, special apps	Mainstream system	Mainstream system
Taking notes or writing	Mainstream system, special apps	Mainstream system	Mainstream system

Given smartphone usage, both cases have reported that around 75–80% of their daily living solutions can be executed using their smartphone. Furthermore, both considered smartphones to be an essential asset in their lives.

## Enablers of Smartphones Use

The participants reported the most essential enabling factors were the presence of screen reader technology, namely, “TalkBack” of Android phones, and the availability of internet data connection of the mobile (Table 2). The participant “UK” quoted that:

**Table 2:** Enabling factors for smartphone use among people with profound visual impairment.

**Factors which help smartphone use**	**Case 1**	**Case 2**
Familiarity of smartphones	Very important (Samsung)	Moderately important (Xiaomi)
Presence of screen reader	Very important (Talkback)	Very important (Talkback)
Availability of third accessible party apps	Very important	Very important
Available Wi-Fi/Data internet at home	Very important	Very important
Power source, including power bank	Less Important	Important
Apps that can read bilingual or multiple languages	Very Important	Important
Basic understanding in English	Important	Moderately Important
Getting help or training on the use of smartphone from others	Important	Very Important
Using headphone	Very Important	Very Important

*The responses were recorded in an ordinal scale: 1: very important, 2: moderately important, 3: important, and 4: less important*.

“Without the screen reader and internet accessibility, our smartphone is like a log lying in a corner of a house.”

Another very important feature is the ability to read multiple languages using the text-to-speech setting of the TalkBack menu. The participant can select a native language, e.g., Hindi, for audio output. This feature is very important because they said many documents, including Hindi, are required to be read in their daily lives. Furthermore, both the cases stated that headphones were one of the very important accessories that help in maintaining their privacy and make less disturbance to a person sitting next to them during travel.

Familiarity with a smartphone is not so important because they said that even if a new one was acquired within 1 or 2 days, they can operate the smartphone comfortably. Finally, both cases underpinned that a supportive environment from peers or family members is important for the initial learning and training of an individual with vision loss.

## Challenges While Using Smartphones

In this report, both cases reported a few most frequent challenges faced by two of them while using smartphones (Table 3). First, poor battery backup of their smartphone, especially during long-hour travel was difficult. Case 1 (UK) stated since they used many accessible apps with voice output, the battery drained out quickly though it was fully charged. Therefore, both cases reported that they always carry power backup devices, such as Power Bank, during the long-hours travel.

**Table 3:** Challenges faced during the smartphone use.

**Challenges**	**Case 1**	**Case 2**
Unlocking the smartphones	Never	Never
Slow screen reader	Sometimes	Sometimes
Screen reader unresponsiveness	Sometimes	Rarely
Third-party accessible app unresponsive	Rarely	Rarely
Not understanding the voice output or sound	Sometimes	Never
Facing problem with the speaker or loudspeaker	Very often	Never
Unintentional selection of icons	Never	Rarely
Getting lost when using a browser	Very often	Rarely
Unable to locate a specific website for a purpose	Rarely	Sometimes
Facing problems for safe keeping	Never	Sometimes
Feeling difficulty in carrying smartphone	Never	Never
Financial constrained to repair maintained phone	Sometimes	Never
Facing issues for poor battery backup	Sometimes	Sometimes
Not a user friendly	Rarely	Never
Difficult to text entry with aloud voice	Very often	Rarely
Confusion due to verbose from smartphone	Sometimes	Sometimes
Disturbance unwanted add or advertisement when using smartphone	Very often	Very often
Smartphone content not readable with screen reader, e.g., CAPCHA	Very often	Very often

*The responses were recorded in an ordinal scale: 1: never, 2: rarely, 3: sometimes, 4: very often, and 5: always*.

Second, typically the loudspeaker is located at the bottom of the smartphone though a smaller speaker is present at the top of the phone that is used for conversations. Such a location of the loudspeaker causes inconvenience to listen to voice output as shared by case 1. Third, the frequent unwanted ads or pop-ups advertisement while using one app also caused a problem in case 2, but not in case 1 since he said that he used an add guard that prevented unwanted ads while using apps. Fourth, few content or labels in the smartphone are not readable with a screen reader. This causes a great challenge to use certain web pages. For example, both cases said that the CAPTCHA and any image files on the page cannot be read by their screen reader. Furthermore, both reported that certain contents were required to be opened by using a mouse or touch screen. This creates a lot of challenges when using smartphones for a certain purpose as shared by cases.

Furthermore, both cases shared that the less frequent issues encountered were slow or unresponsiveness of the screen readers, unable to locate a specific webpage, unable to trace or lose a web page in the middle of browsing, and not accepting voice entry using, such as Google assistant.

There is a paucity of studies available on the technological challenges either hardware or software faced by individuals with visual impairment on smartphone use. However, studies from India reported the most common problem encountered by novice users is language comprehension, including pronunciations and occasional shortcomings in the local language support, challenges in setting up and activation for accessibility features, maintaining the accessibility functionality after reboots, and unable to control the speaking speed while working with text-to-speech (17, 18). Several problems such as operating and using basic accessibility features while interacting touchscreen are also reported in a study conducted in a high-income country (19).

Therefore, the current advanced smartphones still have room for improvement in terms of simplifying the activation process, standardization of the operating procedure for a screen reader, and configuring accessibility features rather than having multiple steps to activate it. For example, several participants of a study conducted in Nepal wanted to have a simple shortcut button for the activation of both built-in and accessible features (10). The same study also concluded that people with visual impairment prefer a fixed region instead of browsing the mobile menu system. Finally, a universal and easy solution to operating for all accessible features could potentially be helpful, particularly to individuals with profound visual impairment.

## Conclusions

The present case report provides valuable insights on how much smartphones enable help in executing daily living solutions in persons with a profound visual impairment. This study gives evidence that without relying on a good visual function, smartphones can help in bringing a wide range of everyday solutions to the visually impaired, and can be concluded that such individuals are one of the potential clients of the current smartphone environment. Furthermore, the smartphone is a universally accepted design without having any stigma on the user. It is, therefore, overarching that such assistive devices should be encouraged to be used so that individuals with vision loss can integrate into society and participate at their best level thereby helping in contributing to their potential.

## Data Availability Statement

The original contributions presented in the study are included in the article/Supplementary Material, further inquiries can be directed to the corresponding author/s.

## Ethics Statement

The studies involving human participants were reviewed and approved by All India Institute of Medical Sciences, New Delhi. The patients/participants provided their written informed consent to participate in this study. Written informed consent was obtained from the individual(s) for the publication of any potentially identifiable images or data included in this article.

## Author Contributions

SS conceived the need for documentation about smartphone use comprehensively among people with severe visual impairment or total blindness, particularly from low- to middle-income countries, further designed the work overall, and wrote the manuscript. SP commented on the draft and edited it. Both authors approved the draft.

## Funding

Indian Council of Medical Research, New Delhi funded (Project Grant no. 2021-6506/F1) this study. The funder does not have any role in the design and conduct of the present study. The investigator performed this study to understand how a person with visual impairment operates a smartphone using various accessible features and apps before the main online survey on the post COVID-19 symptoms starts.

## Conflict of Interest

The authors declare that the research was conducted in the absence of any commercial or financial relationships that could be construed as a potential conflict of interest.

## Publisher's Note

All claims expressed in this article are solely those of the authors and do not necessarily represent those of their affiliated organizations, or those of the publisher, the editors and the reviewers. Any product that may be evaluated in this article, or claim that may be made by its manufacturer, is not guaranteed or endorsed by the publisher.
