# Supplementary material for: Challenges and Enablers for Smartphone Use by Persons With Vision Loss During the COVID-19 Pandemic: A Report of Two Case Studies
Source: Front Public Health. 2022 Jul 6;10:912460. doi: 10.3389/fpubh.2022.912460 (PMC9299074; doi:10.3389/fpubh.2022.912460)
Supplement: Supplementary file 1 [file Data_Sheet_1.PDF]

Purposes, Challenges and Enablers for smartphone Use in persons with visual loss during the COVID-19 pandemic

Date: 10/03/2022

A. General Information of Patient

Name:	S/D/W	Age	Sex	Address *(State)*	*Education: PG, Hindi Lit.
	Chattu Kushwaha	29	1 1) Male 2) Female	Delhi *Contact no:*	Marital status: 2 (Yes=1, No=2)

Occupation 7	BCVA (R/E, in certificate) PL−	BCVA (L/E, in certificate) PL−	Diagnosis (in Certificate): (R) Pthisis (L) Atrophic bulbi

*Occupation code: *1-Govt. Job, 2-Pvt. Job, 3-Shopkeeper, 4-Business, 5-Daily wages, 6- Homemaker, 7-Student*

DISABILITY PERCENTAGE = 100 %

B. Please tell for what purposes do you use smartphone during the COVID-19 pandemic?

	Yes=1, Not in my case=2	Tick/Mention	Enabling factors (Tick)
1. Use for communication	1	Friend ✓, Family members ✓, Teacher ✓,	SMS ✓, Voice call ✓, Audio-visual call ✓,
2. For Health consultation	1	Doctor ✓, Pharmacist ✓, health worker,	SMS, Voice call ✓, Audio-visual call,
3. For getting information on COVID-19	1	About spread ✓/protection ✓/prevention ✓,	Newspaper, Youtube ✓, News channel ✓,
4. Money transfer/payment	1	Family ✓, Friend ✓, Shop ✓, mobile bill/recharge,	Gpay ✓, Phonepe ✓, Paytm ✓,
5. For Online shopping/Tele shopping	1	Clothes, ration, mobile ✓, vegetable, Speaker, Food	Amazon ✓, Flipkart, Blinkit, Bigbasket, Zomato
6. For Reading Print material	2	Medicine/tablet/sanitizer/price tag,	Special apps,
7. For Entertainment	1	Music ✓, movie ✓, TV shows,	Youtube ✓, Netflix, Amazon prime, Tiktok, Wynk, Hotstar
8. For Color identification	1	Red ✓, green ✓, blue ✓,	Special app, Eye-D Pro
9. For Money identification/Handling finance	1	While shopping/keeping ✓/giving ✓ away,	Special app, Mani
10. Use for object identification	2	Shoe, bottle, canes, keys,	Special app,
11. Use for Local transportation/booking	1	Colleges, Market, Hospital, Bank, Railway ✓ station,	OLA ✓, Uber ✓, Rapido, Meru,
12. Indoor navigation (inside the building/home)	2	Bathroom, kitchen, toilet, bedroom, stair, roof,	Special app,
13. Use for taking medicine	2	Identification of medicine, Dose, expiry date,	Special app,
14. Use for Alarm/reminder	1	Getting up ✓, meeting, birthday, Alert	Special app, Alarm
15. Use as Online platform	1	Classes ✓/Meeting ✓/group discussion,	Google meet ✓, Zoom ✓, Microsoft,
16. Use as a Calculator	1	Subtract ✓, addition ✓, divide ✓, multiply ✓,	Special app, Calculator

17. Face recognition	2	Friend, peers, family member,	Special app,
18. Social Media	1	Messaging ✓, talking, chatting ✓,	Facebook ✓, WhatsApp ✓, Instagram ✓, Telegram
19. Photography	1	Documents ✓, Friends, Family members,	Special app, Camera

20. Others if any Mail, Save Contact, Note Pad, Recorder, Call Recording

C. Additional enabling factors that made possible or help you to use smartphones during lockdown/pandemic?

(How frequently do you face/feel it)
1. Very Important 2. Moderately Important 3. Important 4. Not Important at all

	Yes=1, Not in my case=2	Tick/Mention	Tick/Mention			
1. Familiarity/user friendly of smartphones	1	Samsung ✓, Xiaomi, Apple, Vivo, Motorola,	1	2	3 ✓	4
2. Use of screen reader	1	Talkback ✓, Voiceover,	1 ✓	2	3	4
3. Available Wi-Fi/Data internet at home	1	Airtel ✓, Idea, Vodafone, Excitel, hotspot	1 ✓	2	3	4 – Without net phone will [illegible] Box
4. Regular power supplies/Power source at home	1	Inverter, power bank ✓,	1	2	3	4 ✓
5. Apps that can read bilingual/multiple language	1	Vocalizer, Eloquence Text to Speech app ✓,	1 ✓	2	3	4
6. Understanding English	1		1	2	3	4 ✓
7. Getting help from on the use of smartphone	1	Healthcare, Family, Peers ✓,	1	2	3	4 ✓
8. Financial supports from any other agency/NGO	2	Govt. NGO, Pvt. NGO, Semi-govt. NGO, Intl. NGO, Family, Donor	1	2	3	4
9. Using headphone	1	For calling ✓, education ✓, talkback ✓, voiceover,	1 ✓	2	3	4

10. Others if any

During COVID-19 Pandemic

D. Challenges that you encountered, while using smartphone? **(How frequently do you face/feel it)**

	Yes=1, No/Never=2	Tick/Mention
1. At the begging, have you ever felt hesitation or reluctant for smartphone	2	Not applicable / Rarely / Sometimes / very often / always
2. Unlocking the smartphones	2	Not applicable / Rarely / Sometimes / very often / always
3. My screen reader is slow	1	Not applicable / Rarely / Sometimes ✓ / very often / always

4. My special app is unresponsive	2	Not applicable / Rarely / Sometimes / very often / always	
5. Not understanding the meaning of a specific sound	2	Not applicable / Rarely / Sometimes / very often / always	
6. Frequent unintentional selection of icons	1	Not applicable / Rarely / Sometimes / very often / always	
7. Getting lost when using a specific app/icon	1	Not applicable / Rarely / Sometimes / very often / always	
8. Unable to find a specific website	1	Not applicable / Rarely / Sometimes / very often / always	
9. Facing problem for safe keeping (at home/office)	1	Not applicable / Rarely / Sometimes / very often / always	
10. Feeling difficulty in carrying around mobile phone	2	Not applicable / Rarely / Sometimes / very often / always	
11. Shortage of money to repair damaged phone	1	Not applicable / Rarely / Sometimes / very often / always	
12. Facing problem for poor battery backup	1	Not applicable / Rarely / Sometimes / very often / always	– Long Journey
13. Facing problem with the design (not user friendly)	1	Not applicable / Rarely / Sometimes / very often / always	– speaker below
14. Facing problem with my touch screen function	2	Not applicable / Rarely / Sometimes / very often / always	
15. Facing problem with microphone function	1	Not applicable / Rarely / Sometimes / very often / always	
16. Facing problem with speaker/loudspeaker function	1	Not applicable / Rarely / Sometimes / very often / always	
17. Difficult to text entry with aloud voice	1	Not applicable / Rarely / Sometimes / very often / always	
18. Feeling general apps not working properly	1	Not applicable / Rarely / Sometimes / very often / always	
19. Feeling verbose (many voices output) from mobile	1	Not applicable / Rarely / Sometimes / very often / always	
20. Facing unwanted add/advertisement when using app	2	Not applicable / Rarely / Sometimes / very often / always	– Ad Guard
21. Content or labels not readable with a screen reader/special app	1	Not applicable / Rarely / Sometimes / very often / always	– Captcha, Image

22. Others if any Emoji Reader

E. How much smartphones help in doing daily living activities without support from others (*In percentage %*)

........80%.......... % (0-100)

F. How much do you think smartphones is important for your life? | 1 | 1. Very Important 2. Moderately Important 3. Important 4. Not Important at all

Name of Interviewer: VIKA/ Dr. Suraj Singh Senjam

Signature of Interviewer: [illegible]

Purposes, Challenges and Enablers for smartphone Use in persons with visual loss during the COVID-19 pandemic

A. General Information of Patient

Date: 10/03/2022

Name:	S/D/W	Age	Sex	Address *(State)*	*Education:
	Netra Pal	27	2<br>1) Male<br>2) Female	Delhi<br>*Contact no:*	Graduate<br>Marital status: 2 (*Yes=1 No=2*)

Occupation: 1 — Banking

BCVA (R/E, in certificate): PL−

BCVA (L/E, in certificate): 2/60

Diagnosis (in Certificate): (R) Anophthalmia; (L) Pseudophakia c̄ Corneal Opacity

DISABILITY PERCENTAGE = 90% (AIIMS)

*Occupation code: *1-Govt. Job, 2-Pvt. Job, 3-Shopkeeper, 4-Business, 5-Daily wages, 6- Homemaker, 7-Student*

B. Please tell for what purposes do you use smartphone during the COVID-19 pandemic?

	Yes=1, Not in my case=2	Tick/Mention	Enabling factors (Tick)
1. Use for communication	1	Friend ✓, Family members ✓, Teacher ✓,	SMS ✓, Voice call ✓, Audio-visual call ✓,
2. For Health consultation	1	Doctor, Pharmacist, health worker ✓,	SMS, Voice call ✓, Audio-visual call,
3. For getting information on COVID-19	1	About spread ✓/protection ✓/prevention ✓,	Newspaper, Youtube ✓, News channel, Twitter
4. Money transfer/payment	1	Family, Friend ✓, Shop ✓, mobile bill/recharge ✓, Cab	Gpay ✓, Phonepe, Paytm ✓,
5. For Online shopping/Tele shopping	1	Clothes ✓, ration, mobile, vegetable ✓,	Amazon, Flipkart, Blinkit ✓, Bigbasket, Swiggy, Meesho
6. For Reading Print material	1	Medicine ✓/tablet/sanitizer/price tag ✓,	Special apps, Envision AI
7. For Entertainment	1	Music ✓, movie, TV shows,	Youtube ✓, Netflix, Amazon prime,
8. For Color identification	2	Red, green, blue,	Special app,
9. For Money identification/Handling finance	1	While shopping ✓/keeping/giving away ✓,	Special app, Kitna Mani
10. Use for object identification	2	Shoe, bottle, canes, keys,	Special app,
11. Use for Local transportation/booking	1	Colleges, Market, Hospital, Bank ✓, Railway station,	OLA ✓, Uber ✓, Rapido, Meru,
12. Indoor navigation (inside the building/home)	2	Bathroom, kitchen, toilet, bedroom, stair, roof,	Special app,
13. Use for taking medicine	1	Identification of medicine, Dose, expiry date ✓,	Special app, Envision AI
14. Use for Alarm/reminder	1	Getting up ✓, meeting, birthday, Alert, Duty	Special app, Alarm
15. Use as Online platform	1	Classes ✓/Meeting ✓/group discussion,	Google meet ✓, Zoom ✓, Microsoft, CISCO
16. Use as a Calculator	1	Subtract ✓, addition ✓, divide ✓, multiply ✓,	Special app, Calculator with Talkback

		Response	Tick/Mention	Special app
17.	Face recognition	2	Friend, peers, family member,	Special app,
18.	Social Media	1	Messaging ✓, talking ✓, chatting ✓,	Facebook ✓, WhatsApp ✓, Instagram,
19.	Photography	1	Documents ✓, Friends ✓, Family members,	Special app, Camera, Scanner

20. Others if any

C. Additional enabling factors that made possible or help you to use smartphones during lockdown/pandemic?

(How frequently do you face/feel it)

1. Very Important 2. Moderately Important 3. Important 4. Not Important at all

		Yes=1, Not in my case=2	Tick/Mention	Tick/Mention			
1.	Familiarity/user friendly of smartphones ✓	1	Samsung, Xiaomi ✓, Apple, Vivo, Motorola,	1	2 ✓	3	4
2.	Use of screen reader	1	Talkback ✓, Voiceover,	1 ✓	2	3	4
3.	Available Wi-Fi/Data internet at home	1	Airtel, Idea, Vodafone, Excitel, hotspot Jio	1 ✓	2	3	4
4.	Regular power supplies/Power source at home	1	Inverter, power bank ✓,	1	2	3 ✓	4
5.	Apps that can read bilingual/multiple language	1	Vocalizer, Eloquence Text to Speech app ✓,	1 ✓	2	3	4
6.	Understanding English	1		1	2 ✓	3	4
7.	Getting help from on the use of smartphone	1	Healthcare, Family, Peers ✓,	1 ✓	2	3	4
8.	Financial supports from any other agency/NGO	1	Govt. NGO ✓, Pvt. NGO, Semi-govt. NGO, Intl. NGO, Family, Donor	1	2	3	4 - Laptop
9.	Using headphone	1	For calling ✓, education ✓, talkback ✓, voiceover,	1	2 ✓	3	4

10. Others if any

During COVID-19 Pandemic

D. Challenges that you encountered, while using smartphone? **(How frequently do you face/feel it)**

		Yes=1, No/Never=2	Tick/Mention
1.	At the begging, have you ever felt hesitation or reluctant for smartphone	1	Not applicable / Rarely ✓ / Sometimes / very often / always
2.	Unlocking the smartphones	2	Not applicable / Rarely / Sometimes / very often / always
3.	My screen reader is slow	1	Not applicable / Rarely / Sometimes ✓ / very often / always

4. My special app is unresponsive	1	Not applicable / Rarely / Sometimes / very often / always
5. Not understanding the meaning of a specific sound	2	Not applicable / Rarely / Sometimes / very often / always
6. Frequent unintentional selection of icons	1	Not applicable / Rarely / Sometimes / very often / always
7. Getting lost when using a specific app/icon	1	Not applicable / Rarely / Sometimes / very often / always
8. Unable to find a specific website	1	Not applicable / Rarely / Sometimes / very often / always
9. Facing problem for safe keeping (at home/office)	1	Not applicable / Rarely / Sometimes / very often / always
10. Feeling difficulty in carrying around mobile phone	2	Not applicable / Rarely / Sometimes / very often / always
11. Shortage of money to repair damaged phone	2	Not applicable / Rarely / Sometimes / very often / always
12. Facing problem for poor battery backup	1	Not applicable / Rarely / Sometimes / very often / always
13. Facing problem with the design (not user friendly)	2	Not applicable / Rarely / Sometimes / very often / always
14. Facing problem with my touch screen function	1	Not applicable / Rarely / Sometimes / very often / always
15. Facing problem with microphone function	1	Not applicable / Rarely / Sometimes / very often / always
16. Facing problem with speaker/loudspeaker function	2	Not applicable / Rarely / Sometimes / very often / always
17. Difficult to text entry with aloud voice	1	Not applicable / Rarely / Sometimes / very often / always
18. Feeling general apps not working properly	1	Not applicable / Rarely / Sometimes / very often / always
19. Feeling verbose (many voices output) from mobile	1	Not applicable / Rarely / Sometimes / very often / always
20. Facing unwanted add/advertisement when using app	1	Not applicable / Rarely / Sometimes / very often / always
21. Content or labels not readable with a screen reader/special app	1	Not applicable / Rarely / Sometimes / very often / always

22. Others if any ____________________

E. How much smartphones help in doing daily living activities without support from others (*In percentage %*)

75-80 .... % (0-100)

F. How much do you think smartphones is important for your life? [1] 1. Very Important 2. Moderately Important 3. Important 4. Not Important at all

Name of Interviewer: VIKAS **Signature of Interviewer:** [signature]
